# Neopterin Concentration in Umbilical Cord Blood as a Reflection of Maternal Insulin Resistance—A Pilot Study

**DOI:** 10.3390/biology14091157

**Published:** 2025-09-01

**Authors:** Aleksandra Chęcińska-Kopeć, Ewa Pruszynska-Oszmalek, Zuzanna Checinska-Maciejewska, Anna Rekas-Dudziak, Małgorzata Wojciechowska, Piotr Ślósarz, Hanna Krauss, Krzysztof Szymanowski, Pawel A. Kolodziejski

**Affiliations:** 1 Obstetrics and Gynecology Department, Holy Family Hospital, 60-235 Poznań, Poland; 2 Department of Animal Physiology, Biochemistry and Biostructure, Faculty of Veterinary Medicine and Animal Science, Poznań University of Life Sciences, Wolynska Street 35, 60-637 Poznań, Poland; ewa.pruszynska@up.poznan.pl; 3 Department of Medicine, Calisia University, 62-800 Kalisz, Poland; zuchecinska@gmail.com (Z.C.-M.); hjk12@poczta.fm (H.K.); 4 Gynaecological-Obstetric Clinical Hospital of the Poznań University of Medical Sciences, 60-535 Poznań, Poland; annarekasdudziak@gmail.com; 5 Anesthesia Intensive Care Pain Medicine, MSWiA Hospital, 60-631 Poznan, Poland; 6 Department of Mother and Child Health, Poznan University of Medical Sciences, 61-701 Poznań, Poland; malgorzata59@onet.eu; 7 Department of Animal Breeding and Animal Product Quality Assessment, Faculty of Veterinary Medicine and Animal Science, Poznań University of Life Sciences, Słoneczna 1, 62-002 Złotniki, Poland; piotr.slosarz@up.poznan.pl; 8 Department of Perinatology and Gynecology, Poznan University of Medical Sciences, 61-701 Poznań, Poland; kp.szymanowski@wp.pl

**Keywords:** neopterin, insulin resistance, umbilical cord blood, maternal metabolism

## Abstract

Background/Objective: Insulin resistance (IR) during pregnancy, even in women with normal body mass index (BMI), may affect maternal and fetal metabolic and immune status. This study aimed to evaluate neopterin (NPT), leptin, insulin, and ghrelin concentrations in maternal blood (MB) and umbilical cord blood (CB) in normoglycemic women with and without IR, all with normal BMI. Methods: Peripheral and cord blood was collected from 36 Caucasian women with term, uncomplicated vaginal deliveries. The participants were classified into control (*n* = 16; age = 30.81 ± 4.875 years) and IR (*n* = 20; age = 31.95 ± 4.979 years) groups based on a professional medical diagnosis. Anthropometric parameters were recorded, and metabolic/hormonal markers were measured using ELISA and RIA. Results: NPT concentrations in CB were significantly higher in the IR group (*p* < 0.05), correlated positively with MB NPT levels (r = 0.3809, *p* < 0.05). A significantly higher concentration of both insulin and leptin was observed in the MB of women with IR compared to the control group (*p* < 0.0001), whereas in CB, only insulin concentration was significantly higher in the IR group than in healthy controls (*p* < 0.05). Ghrelin levels did not differ between the groups. Conclusions: Insulin resistance in non-obese pregnant women is associated with increased NPT concentration in CB, which may suggest fetal immune activation. However, defining the role of NTP as a metabolic “messenger” between mother and child requires further study.

## Simple Summary

Insulin resistance (IR), even in women with normal BMI, may influence both maternal and fetal health during pregnancy. In this study, we compared metabolic and hormonal markers in maternal (MB) and umbilical cord blood (CB) from women with and without IR. We found higher neopterin levels in the CB of the IR group, along with increased maternal insulin and leptin, and higher cord blood insulin. These results suggest that IR in non-obese pregnant women may be linked to fetal immune activation, warranting further investigation.

## 1. Introduction

In recent years, there has been growing interest in maternal metabolic imprinting as a potential factor influencing metabolic programming and the risk of developing metabolic disorders later in a child’s life [1,2,3]. Previous studies have demonstrated associations between various maternal and neonatal metabolic parameters—reflected, for example, in cord blood—and neonatal anthropometric outcomes, as well as indicators of metabolic imprinting. Notably, maternal metabolic syndrome has been shown to influence infant development, in part through altered insulin signaling, a key regulator of energy homeostasis [4]. In parallel, recent studies have highlighted the role of the immune system and its mediators in the etiology of metabolic diseases, including type 2 diabetes and obesity-related insulin resistance (IR). Immune-derived factors produced by the maternal organism have been shown to cross the placental barrier and potentially modulate metabolic programming in the offspring [5,6,7]. This raises the question of whether factors present in maternal blood, capable of crossing the placenta into umbilical cord blood, may lead to long-term metabolic alterations. However, before this question can be answered, it is essential to establish whether these biological factors are detectable in umbilical cord blood. The umbilical cord and cord blood serve as a critical intermediary connection between the mother and fetus, facilitating the exchange of nutrients, gases, and signaling molecules. Cord blood contains various metabolic, hormonal, and immune factors that reflect both maternal physiology and fetal responses. As such, it may serve as a valuable indicator of maternal–fetal interactions and the influence of maternal conditions on fetal development [8,9].

One such maternal pathological condition is insulin resistance. This is particularly important not only due to the critical role of insulin in fetal development, but also because maternal insulin resistance is linked to metabolic dysregulation in the mother, which may have multiple downstream effects [10]. Studies in humans and rodents suggest that maternal glucose metabolism disorders, such as insulin resistance or diabetes, increase the risk of impaired glucose tolerance and obesity in offspring later in life. The results demonstrated that maternal insulin resistance and hyperinsulinemia impair endocrine pancreas development and lead to metabolic disturbances in offspring, including higher postnatal glucose, insulin, leptin, glucagon, and GLP-1 levels, along with reduced β-cell area and proliferation [11,12]. Moreover, it has been known for many years that both IR and diabetes are treated as chronic inflammatory conditions due to the metabolic consequences they carry [13]. This is due to the multitude of factors that are produced during the immune system reaction, some of which can also cross the placental barrier.

One of the compounds produced as an element of the immune response is neopterin, whose role has not yet been investigated in the context of its association with the phenomenon of insulin resistance and maternal imprinting, but whose participation in the development of insulin resistance has been demonstrated in adults [14,15]. Neopterin (NPT) is a sensitive marker of nonspecific cellular immune activation [16]. It belongs to a group of pteridines—a family of nitrogen-containing heterocyclic compounds, some of which act as pigments that are widely distributed. Neopterin is synthesized by monocytes, macrophages, and dendritic cells in response to stimulation by interferon-gamma (IFN-γ), which is primarily secreted by CD4⁺ and CD8⁺ T lymphocytes as well as natural killer (NK) cells [17,18]. Although its role is mainly limited to the immune response, there are indications that it may also affect echogenic homeostasis, e.g., in gestational diabetes, where higher maternal and cord blood neopterin concentration was observed, which was negatively correlated with Apgar scores [19,20].

Therefore, using a traditional retrospective case-control study design, we aimed to investigate whether NPT concentrations differ in the maternal and cord blood of pregnant women with insulin resistance compared to healthy controls. Such differences may suggest that alterations in NPT levels in cord blood are associated with the potential development of metabolic disorders in offspring and may indicate its involvement in metabolic imprinting. Additionally, we examined whether NPT levels correlate with other hormonal factors—such as insulin [21], leptin [22], or ghrelin [23,24]—which play key roles in the pathogenesis of insulin resistance.

## 2. Materials and Methods

### 2.1. Study Participants and Ethics

The study was conducted on Caucasian women admitted to the Gynaecologic and Obstetrical University Hospital in Poznań for planned delivery. All procedures were carried out in accordance with the principles of the Declaration of Helsinki. The study protocol received approval from the Clinical Research Ethics Committee of the Poznan University of Medical Sciences (approval number 1043/13). All women participating in the study provided written informed consent. The study was conducted between 2015 and 2018. The study material consisted of maternal peripheral blood (MB) and umbilical cord blood (CB) collected on the day of delivery from healthy, normal-weight women (BMI 17.8–24.9 kg/m^2^) and from women diagnosed with insulin resistance who also had a normal BMI. In two cases, based on the physician’s subjective assessment of body composition, women with a muscular build and BMI values of 25.38 and 25.56 were included in the insulin resistance group. The group allocation was based on a professional clinical diagnosis, which included a comprehensive assessment of the patient’s medical history performed by a qualified physician in conjunction with commonly accepted diagnostic parameters. This approach was intended to ensure clinically relevant classification, particularly in cases where isolated biochemical indices might be influenced by transient physiological or environmental factors. The HOMA-IR index was also used as part of the diagnostic process, with a threshold value of 2.5 indicating potential insulin resistance. The inclusion criteria were the absence of pregnancy complications and delivery via spontaneous vaginal birth.

Exclusion criteria, consistent with those applied in our previous studies (Wojciechowska et al. [25]), were also adopted in the present study. Maternal anthropometric parameters, including height, pre-pregnancy body weight, BMI, and body weight on the day of delivery, and neonatal somatic features—including body length, head and chest circumference, and birth weight—were measured according to standard procedures. The data are presented in Table 1 and Table 2.

### 2.2. Umbilical Cord Blood (UCB) and Maternal Blood (MB) Collection

Maternal blood (MB) and umbilical cord blood (CB) were collected using BD Vacutainer^®^ SST™ II Advance tubes (BD Diagnostics, NJ, USA). The samples were allowed to clot at room temperature for 15–20 min and then centrifuged at 3500× *g* for 12 min at 4 °C. The resulting serum was aliquoted into 0.5 mL tubes and stored at −80 °C until analysis.

### 2.3. Metabolic and Hormonal Profile

In maternal and umbilical cord serum, the concentrations of neopterin, ghrelin, leptin, and insulin were measured. Serum concentration of NPT was measured by means of immunoenzymatic assay (ELISA; ELItest Neopterin catalog no. 99.1 and 95.4, BRAHMS, Hennigsdorf, Germany). Total and active plasma ghrelin concentration was measured by radioimmunoassay (RIA) using the Ghrelin (Total) RIA Kit and Ghrelin (Active) RIA Kit (catalog no. GHRT-89HK and GHRA-88HK, Millipore, Burlington, MA, USA). Leptin levels were measured by sandwich ELISA using the DRG Leptin ELISA Kit (catalog no. EIA-2395, DRG Instruments GmbH, Marburg, Germany). Serum insulin concentrations were measured using sandwich ELISA with the DRG Insulin ELISA Kit (catalog no. EIA-2935, DRG Instruments GmbH, Germany). Glucose concentration was measured using a commercially available colorimetric kit from Pointe Scientific. The optical density of samples from colorimetric and ELISA assays was measured using a Synergy 2 Microplate Reader (BioTek, Winooski, VT, USA). Gamma radiation from the RIA samples was quantified with a Wallac Wizard 1470 Gamma Counter (PerkinElmer, Waltham, MA, USA).

### 2.4. Statistical Analysis

Statistical analyses were performed using an unpaired two-tailed Student’s *t*-test or the Mann–Whitney U test, depending on data distribution. The normality of distribution in each group was assessed using the D’Agostino & Pearson test. Statistical significance was defined as *p* < 0.05 (*) and *p* < 0.01 (**). Correlations between serum NPT concentrations and other parameters were evaluated using Pearson’s correlation and linear regression analyses, with significance set at *p* < 0.05. Exact *p*-values are reported in the Results section, where applicable. Given the relatively small sample size and the limited number of statistical comparisons, no *p*-value adjustment was applied. All analyses were performed using GraphPad Prism 10 (GraphPad Software, San Diego, CA, USA.

## 3. Results

### 3.1. Anthropometric Metabolic Parameters

The analysis of maternal anthropometric parameters—including pre-pregnancy weight, height, BMI, and weight at delivery—revealed no statistically significant differences between the groups. A statistically significant difference was observed in the insulin resistance index (HOMA-IR) between healthy individuals and those with insulin resistance (*p* < 0.01; Table 1). No statistically significant differences were found in the neonatal parameters measured (Table 2).

### 3.2. NPT Concentration Changes

**Figure 1:**
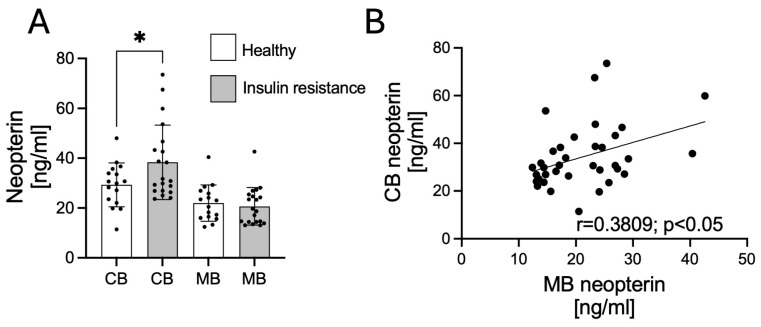
Changes in NPT concentration in maternal and umbilical cord blood in healthy and insulin-resistant mothers (**A**). Values are presented as mean ± standard deviation of the mean. Statistically significant differences between the means are marked * *p* < 0.05. Correlations between NPT levels in mother’s blood and umbilical cord blood (**B**). The r-value indicates a correlation, and the *p*-value indicates the significance of the correlation. MB—mother’s blood; CB—umbilical cord blood.

NPT concentrations were measured in MB and CB from healthy and insulin-resistant subjects. An increase in NPT concentration in CB (*p* < 0.05) of insulin-resistant subjects compared to healthy subjects was demonstrated (Figure 1A). We also noted the positive correlation of the NPT levels in CB and MB (r = 0.3809, *p* < 0.05, Figure 1B).

### 3.3. NPT, Leptin, Insulin, and Ghrelin

**Figure 2:**
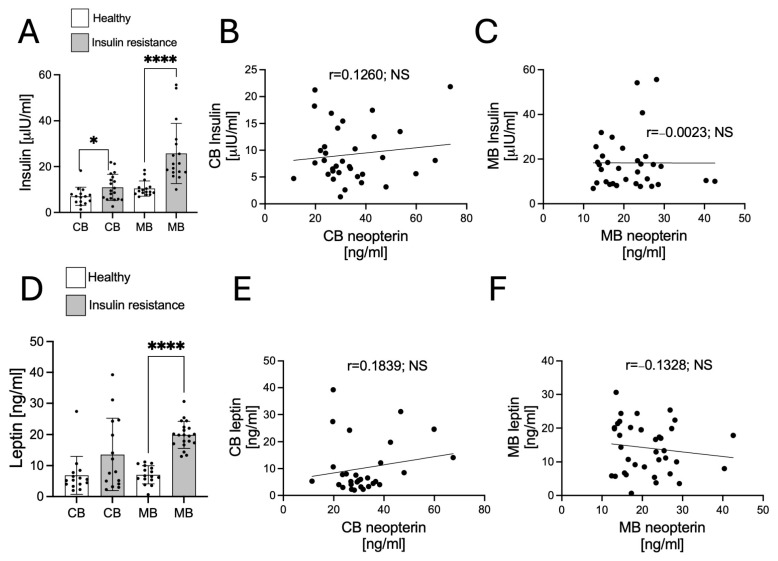
Changes in insulin (**A**) and leptin (**B**) concentrations in umbilical cord blood (CB) and maternal blood (MB) from healthy and insulin-resistant subjects. Values are presented as mean ± standard deviation (SD). Statistically significant differences between the means of the studied groups are indicated as follows: * *p* < 0.05; **** *p* < 0.0001. Correlations between neopterin (NPT) and insulin in CB and MB are shown in panels (**C**,**D**), while correlations between NPT and leptin are presented in panels (**E**,**F**), respectively. The r-value represents the strength of the correlation, and the *p*-value denotes its statistical significance. NS–not significant.

**Figure 3:**
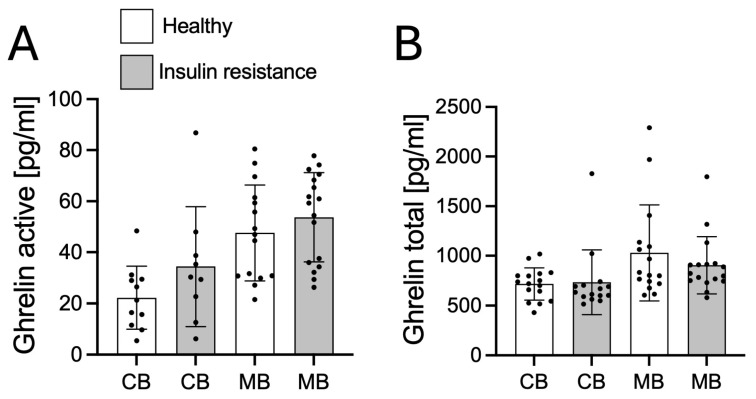
Changes in active (**A**) and total (**B**) ghrelin concentrations in CB and MB from healthy and insulin-resistant subjects. Values are presented as mean ± SD of the mean.

Next, we investigated the changes in insulin, leptin, and ghrelin in CB and MB and their possible correlation with CB and MB NPT. The study demonstrated significantly higher concentrations of insulin in both umbilical cord blood (CB) (Figure 2A; *p* < 0.05) and maternal blood (MB) (Figure 2A; *p* < 0.0001) in the insulin-resistant group compared to the healthy group. Subsequently, we investigated whether these differences were associated with neopterin concentrations in CB (Figure 2B) and MB (Figure 2C). Pearson correlation analysis revealed no statistically significant relationship between neopterin and insulin concentrations in either CB or MB. The results regarding changes in serum leptin concentrations revealed a statistically significant increase in maternal blood (MB) in the insulin resistance group compared to the healthy group (Figure 2D; *p* < 0.0001). In umbilical cord blood (CB), no statistically significant differences were observed. Analysis of the relationship between leptin and neopterin levels in CB and MB showed no statistically significant correlation between leptin and neopterin concentrations. Similarly, the analysis of the active and total ghrelin concentrations revealed no significant differences in CB or MB between the studied groups. Moreover, no correlation was found between ghrelin levels and neopterin concentrations (Figure 3A,B).

## 4. Discussion

Insulin resistance is a physiological adaptation that occurs during pregnancy to ensure adequate glucose supply to the developing fetus [26]. However, when this mechanism becomes excessive or dysregulated (for example, when insulin resistance is present in the mother before pregnancy), it may contribute to gestational diabetes mellitus (GDM) and is associated with an increased risk of adverse maternal and neonatal outcomes, including macrosomia, preeclampsia, and long-term metabolic disorders in the offspring [27,28]. Early identification of metabolic disturbances and their impact on the intrauterine environment is essential for guiding prenatal care and mitigating long-term consequences.

That is why, for many years, the influence of maternal and/or paternal factors on the future metabolic health of offspring has been widely discussed. Numerous mechanisms have been identified that shape a child’s development and are influenced by metabolic alterations in the maternal organism, including obesity, hyperglycemia, and nutritional stress, among others [29,30].

Increasing attention is being paid to changes in the concentrations of biologically active substances, such as hormones, peptides, and pterins, that can cross the placental barrier and impact both fetal development and the programming of the child’s future metabolism [31,32]. One of them is neopterin, which we studied. Our findings demonstrated a correlation between neopterin concentrations in cord blood serum and maternal blood serum. We also found that neopterin levels were higher in cord blood collected from patients with insulin resistance. Additionally, our results showed a weak correlation between leptin and neopterin concentrations, while no significant association was found between neopterin and either insulin or ghrelin levels.

The relationship between neopterin concentrations in maternal and fetal blood serum was first described nearly 30 years ago [33,34]. One of the earliest studies on this peridine-derived substance, published in 1999, demonstrated that fetal neopterin levels increase significantly throughout gestation and are higher than those in maternal blood. However, the study did not find a statistically significant correlation between maternal and fetal neopterin levels, reporting only a trend (*p* = 0.07) [34]. Additionally, researchers observed significantly elevated neopterin concentrations in cases of intra-amniotic infection and severe preeclampsia, suggesting a possible role for neopterin in activating the fetal cellular immune response [34]. Given the role of this substance in immune responses, most studies on NPT from that period focused on changes in its concentration during viral infections, including HIV, HTLV-I, and the herpes simplex virus [35,36,37]. At the same time, research has begun to explore the role of neopterin in infections in the context of metabolic disorders associated with obesity and insulin resistance [14]. These studies revealed, among other findings, that elevated neopterin levels are observed in individuals with increased blood glucose concentrations and higher body mass [14]. These changes have been linked to the immune stimulation that occurs in obesity. As we now know, this pathophysiological condition should be considered an inflammatory condition of the body. Elevated neopterin concentrations in individuals with carbohydrate–lipid metabolism disorders—such as obesity, diabetes, metabolic syndrome, PCOS, and related conditions—have been repeatedly confirmed in subsequent studies [38,39,40]. Interestingly, in the majority of these studies, elevated neopterin concentrations were correlated with a higher body weight in patients. Our studies presented in this paper seem all the more important, as they indicate increased neopterin concentration in the umbilical cord blood of newborns born to mothers with insulin resistance. This result suggests disturbances in the activation of fetal cellular immunity in conditions of maternal metabolic disorders, even if they are not accompanied by obesity, because NPT, as a product of macrophages activated by interferon γ, is considered an early biomarker of immune system activity.

Another relationship that was analyzed was the association between neopterin (NPT) and hormones involved in the regulation of food intake and carbohydrate-lipid metabolism—insulin, secreted by the pancreas, and leptin, produced by adipose tissue and ghrelin mainly secreted by the GI tract. The analysis revealed no correlation between insulin and neopterin, which may suggest that their secretion is regulated through independent mechanisms. It is also worth noting that although insulin has been shown—particularly in animal models—to exert indirect effects on the immune system [41], the available literature does not indicate any direct relationship between these substances [17], which was also proved by our results. The study also observed significantly higher leptin concentration in the blood of the mother with insulin resistance, while there were no differences in cord blood. Additionally, we did not observe any correlation between leptin and neopterin. Previous studies on the relationship between leptin and NPT have shown that concomitant changes in the concentration of these substances depend primarily on the type of disorder that may affect the concentration of both compounds. For example, a positive correlation between NPT and leptin was demonstrated in obese adolescents with NAFLD [42], while no such correlation was demonstrated in patients with rheumatoid arthritis [43]. However, these changes may be explained by the immunomodulatory effect of leptin on obesity. Numerous studies have shown that leptin can influence the promotion of B cell production, reduce their apoptosis, and also modulate the work of other cells/factors of the immune system, which may explain this connection [44]. However, this was observed mainly in adolescents with fatty liver disease, although pregnancy as a specific metabolic condition did not show such correlations [42].

Analysis of active and total ghrelin levels did not show significant differences between groups or correlations with NPT levels, which is confirmed by some previous studies suggesting variability of ghrelin levels depending on metabolic state and gestational age [45]. However, as in the case of insulin, the lack of correlation between these substances has also been described in the literature [42]. Low neopterin levels generally indicate the absence of significant Th1-type immune activation and are typically observed in healthy individuals without ongoing infections or inflammatory processes. In some cases, low concentrations may also reflect immunosuppression; for example, due to pharmacological treatment, certain immune deficiencies, or antiretroviral therapy, which is associated with a decrease in NPT [46]. In the context of pregnancy, low neopterin levels may suggest the absence of pathological immune activation, which is favorable for normal fetal development. In light of our findings and the existing literature, alterations in NPT concentration may serve as a useful indicator for diseases involving immune system activation. However, further in-depth research is needed to explore its potential long-term effects in infants and children.

Although our study is the first to report changes in neopterin concentrations in serum and cord blood from mothers with insulin resistance and normal body weight, we acknowledge many limitations. The most significant is the small sample size, which limits the generalizability of our findings. Additionally, due to the limited amount of research material, we were unable to repeat the tests that did not give results, which in several cases also narrowed the number of observations in the study groups. Moreover, due to restricted access to biological material and its limited quantity, we focused on a select number of indicators and their correlations, which in our opinion should be improved. Nonetheless, we believe that our results provide valuable insights and underscore the need for further research to better understand the maternal–fetal relationship, particularly in the context of maternal imprinting and its potential influence on the child’s metabolic and/or immune system programming.

## 5. Conclusions

In conclusion, the findings suggest that insulin resistance in pregnant women—even those with a normal BMI—may affect the fetal immune environment, as indicated by increased neopterin levels in cord blood. This may have important implications for the postnatal development of a child’s immune system. However, these associations should be validated in larger cohorts with long-term follow-ups.

## Figures and Tables

**Table 1:** Characteristics of mothers.

PARAMETER	Control (*n* − 16)	Insulin Resistance (*n* − 20)
Before Pregnecy		
Age (years)	30.81 ± 4.875	31.95 ± 4.979
Height (cm)	166.7 ± 5.952	168.0 ± 8.204
Body mass (kg)	58.94 ± 6.027	64.10 ± 9.369
BMI (kg/m^2^)	21.22 ± 1.939	22.63 ± 2.103
HOMA-IR	1.988 ± 0.705	4.175 ± 1.445 **
On The Day Of Birth		
Body mass (kg)	74.56 ± 9.208	78.75 ± 12.53

Values are presented as mean ± SD. Statistically significant differences between healthy vs. insulin-resistant patients are marked for *p* < 0.01 (**). For parameters of age, height, body mass, and BMI, a two-tailed Student’s *t*-test was used, whereas for HOMA-IR, the Mann–Whitney U test was applied.

**Table 2:** Anthropometric parameters of newborns.

PARAMETER	Non Obese	Obese
Child gender (M/F)	6/10	9/11
Head circumference (cm)	35.06 ± 0.981	35.40 ± 1.818
Chest circumference (cm)	34.50 ± 2.098	34.75 ± 2.124
Abdominal circumference (cm)	34.81 ± 1.870	34.03 ± 3.827
Thigh circumference (cm)	13.69 ± 1.352	14.35 ± 1.226
Arm circumference (cm)	12.69 ± 1.991	12.05 ± 0.945
Body mass (kg)	3.364 ± 0.308	3.419 ± 0.348

Values are presented as mean ± SD. For parameters of body mass, a two-tailed Student’s *t*-test was used, whereas for other parameters, the Mann–Whitney U test was applied.

## Data Availability

The data presented in this study are available on reasonable request from the corresponding author.
